# MicroRNA profiling in canine multicentric lymphoma

**DOI:** 10.1371/journal.pone.0226357

**Published:** 2019-12-11

**Authors:** Karlee K. L. Craig, Geoffrey A. Wood, Stefan M. Keller, Anthony J. Mutsaers, R. Darren Wood

**Affiliations:** 1 Department of Pathobiology, Ontario Veterinary College, University of Guelph, Guelph, Ontario, Canada; 2 Department of Clinical Studies, Ontario Veterinary College, University of Guelph, Guelph, Ontario, Canada; Centre de Recherche en Cancerologie de Lyon, FRANCE

## Abstract

Lymphoma is the most common hematopoietic tumour in dogs and is remarkably similar to the human disease. Tumour biomarker discovery is providing new tools for diagnostics and predicting therapeutic response and clinical outcome. MicroRNAs are small non-coding RNAs that participate in post-transcriptional gene regulation and their aberrant expression can impact genes involved in cancer. The aim of this study was to characterize microRNA expression in lymph nodes and plasma from dogs with multicentric B or T cell lymphoma compared to healthy control dogs. We further compared expression between lymph nodes and corresponding plasma samples and assessed changes in expression at relapse compared to time of diagnosis. Lastly, we investigated microRNAs for association with clinical outcome in patients treated with CHOP chemotherapy. A customized PCR array was utilized to profile 38 canine target microRNAs. Quantification was performed using real time RT-qPCR and relative expression was determined by the delta-delta Ct method. In lymph nodes, there were 16 microRNAs with significantly altered expression for B cell lymphoma and 9 for T cell lymphoma. In plasma, there were 15 microRNAs altered for B cell lymphoma and 3 for T cell lymphoma. The majority of microRNAs did not have correlated expression between lymph node and plasma and only 8 microRNAs were significantly different between diagnosis and relapse. For B cell lymphoma, 8 microRNAs had differential expression in the non-remission group compared to dogs that completed CHOP in complete remission. Four of these microRNAs were also altered in patients that died prior to one-year. Kaplan-Meier survival curves for high versus low microRNA expression revealed that 10 microRNAs were correlated with progression-free survival and 3 with overall survival. This study highlights microRNAs of interest for canine multicentric lymphoma. Future goals include development of microRNA panels that may be useful as biomarkers with the intent to provide improved outcome prediction to veterinary cancer patients.

## Introduction

Lymphoma is the most common hematopoietic tumour in dogs and displays significant clinical and pathological overlap with the human disease [1]. The predominant form of presentation is multicentric, which is characterized by generalized lymphadenopathy [2]. This form of lymphoma in dogs is routinely diagnosed by cytologic examination of affected lymph nodes. Further characterization may be pursued for immunophenotyping, grading and clinical staging, all of which may assist in predicting biological behavior.

Lymphoma is a systemic disease that is frequently treated with multi-agent chemotherapy. Remission with chemotherapy is often achieved, but the prognosis is variable, and it is not possible to conclude with certainty which dogs will respond to therapy and for how long.

MicroRNAs (miRNAs) are small non-coding RNA molecules of approximately 22 nucleotides in length that participate in post-transcriptional gene regulation to modify biological processes [3]. Dysregulation of miRNA may affect target genes involved in cancer with functional roles in initiation, progression and metastasis [4]. The widespread presence of miRNA in cells, tissues and body fluids combined with their robust stability may represent an extensive minimally-invasive resource of biomarkers [5].

Cancer-specific miRNA profiles can be created by identifying miRNAs with significantly altered expression between diseased and healthy individuals. miRNAs with prognostic significance may further be used as predictors of various disease outcomes. Multiple miRNAs have been recognized in predicting complete remission and response rates, progression-free survival and overall survival for human patients with diffuse large B cell lymphoma (DLBCL). A miRNA-cell of origin (COO) signature composed of 8 miRNAs has been characterized to differentiate between the more aggressive activated B cell (ABC) subtype and the germinal centre B cell (GCB) subtype and a separate panel of 9 miRNAs was established for prognosis [6].

For canine lymphoma, there are limited studies exploring aberrant miRNA expression and association with prognostic information. Uhl et al (2011) profiled canine lymphoid cell lines and clinical lymph node samples from high-grade lymphoma cases using a modified Cancer miRNA qPCR array for 95 different human miRNAs [7]. In B cell lymphomas, they found increased expression of miR-19a/19b and decreased expression of miR-181a, miR-203, miR-204 and miR-218. In T cell lymphomas, there was increased expression of miR-17-5p, miR-20a, miR-106a, miR-181b and miR-183 and decreased expression of miR-149, miR-186 and miR-218. Two additional studies profiled four specific miRNAs (miR-17-5p, miR-29b, miR-155, miR-181a) using single tube human Taqman assays to investigate canine lymphoma. Mortarino et al (2010) profiled lymph node samples and detected significant upregulation of miR-17-5p in B cell lymphoma and miR-181a in T cell lymphoma [8]. There was a significant difference in the miR-181a/miR-17-5p gene molar ratio between B cell and T cell lymphomas, but it did not distinguish between high and low-grade lymphomas based on the updated Kiel classification. Albonico et al (2013) investigated biopsies from primary splenic lymphoma and found that the miR-17-5p/miR-155 molar ratio correlated with grade according to the WHO classification system [9].

When miRNAs are overexpressed in cancer they can function as “oncomiRs” by downregulating tumour suppressor genes or genes that control cell differentiation or apoptosis. Conversely, some miRNAs function as tumour suppressors that usually prevent tumor development by downregulating oncogenes [10]. Underexpression of these tumour suppressor miRNAs results in lack of oncogene inhibition resulting in promotion of tumourigenesis. Recognition of miRNAs that act as drivers for the development of lymphoma may aid in future miRNA-targeted therapy or in restoration of physiological levels as a new strategy for individualized treatment [11].

The objectives achieved in our study were to: 1) characterize the miRNA profiles in lymph node and plasma samples from dogs with lymphoma compared to healthy control dogs, 2) compare profiles from lymph node aspirates with corresponding plasma samples, 3) compare profiles at diagnosis with paired samples at relapse, and 4) assess profiles for associations with clinical outcome including response to CHOP (cyclophosphamide, doxorubicin, vincristine, prednisone) chemotherapy, one-year survival, progression-free survival (PFS) and overall survival (OS).

## Materials and methods

### Case recruitment

Informed consent was obtained from all clients and an animal utilization protocol (AUP) was approved by the University of Guelph Animal Care Committee (Protocol Number: 3815).

To assess baseline miRNA expression, lymph node and plasma samples were collected from 10 healthy control dogs. We obtained informed owner consent for 8 pets and kennel consent for 2 shelter dogs. Inclusion criteria included no abnormalities on physical examination, complete blood count, biochemistry profile and lymph node cytology. Dogs with known history of disease or an abnormal laboratory result were excluded.

Strict inclusion and exclusion criteria were applied for selection of dogs with lymphoma referred to the Animal Cancer Center of the Ontario Veterinary College (OVC) Health Sciences Centre. Only those with newly diagnosed multicentric lymphoma were considered. Cases had to be free of concurrent disease and naïve with respect to prednisone therapy. Cytology was required for standard diagnosis and immunophenotyping by flow cytometry had to be performed to confirm B or T cell origin. Dogs with other forms of lymphoma or concurrent disease at time of diagnosis were excluded. Only patients that received CHOP chemotherapy (25-week protocol) were included in the assessment for clinical outcomes [12].

### Immunophenotyping

Lymph node aspirates were collected into flow cytometry buffer and were analyzed immediately or stored at 4°C for a maximum of 24 hours. The flow buffer was composed of phosphate buffered saline (PBS) with 1% horse serum, 5mM ethylenediaminetetraacetic acid (EDTA) and 0.1% sodium azide. Flow cytometric immunophenotype of the neoplastic cells was determined using a FACScan instrument (BD Biosciences, Mississauga, Canada). Leukocytes were stained with a panel of 11 antibodies including: CD3_FITC_ (CA17.2A12, T lymphocytes), CD4_FITC_ (YKIX302.9, neutrophils and T-helper lymphocytes), CD5_FITC_ (YK1X322.3, T lymphocytes), CD8α_PE_ (YCATE55.9, cytotoxic T lymphocytes), CD14_PE_ (TUK4, monocytes), CD18_A647_ (CA1.4E9, all leukocytes), CD21_PE_ (Ca2.1D6, B lymphocytes), CD22_PE_ (RFB4, Novus, B lymphocytes), CD34_PE_ (1H6, hematopoietic stem cells), CD45_FITC_ (YK1X716.13, all leukocytes) and MHCII (YK1X334.2, lymphocytes and monocytes) (Bio-Rad, Berkeley, CA, USA).

### Target miRNA selection

A pilot study based on pooled groups composed of thirty dogs in total was completed to help design a custom PCR array to be utilized for this study. The goal was to identify miRNAs with robust amplification and differential expression between dogs with lymphoma and healthy controls. An array-based model (384-well format) known as the dog miRNome miScript miRNA PCR array (Qiagen, Toronto, Canada) was used to profile 277 miRNA sequences in the canine genome. A greater than 5-fold increase or decrease in expression was deemed significant for purposes of the pilot study. This definition was chosen to considerably narrow down the number of target miRNAs to 58 miRs. Furthermore, only miRNAs with an average Ct value less than 30 (high amplification) for each group were considered for the customized PCR array. This criterion was used to increase the probability of detection in all subsequent samples and further narrowed it down to 40 miRs. The ultimate selection of miRNA sequences for the custom array was based on findings of the pilot study, review of the literature and inclusion of commonly used endogenous controls.

The customized miScript miRNA PCR array (Qiagen) was then used to quantify expression of 38 canine miRNAs (miRs) in lymph node and corresponding plasma samples. A summary of the target miRNAs and controls are listed in Table 1. The mature sequence for each miRNA can be accessed through GeneGlobe (Qiagen, www.qiagen.com).

**Table 1:** Customized canine target miRNAs and controls.

Custom PCR array	
**Canine target miRs**	cfa-miR-15a, -16, -18a, -19a, -19b, -21, -23a, -26b, -27a, -29a, -29b, -29c, -30b, -31, -34a, -99a, -101, -125a, -125b, -127, -130b, -143, -145, -146a, -148a, -150, -155, -181a, -181b, -181c, -181d, -182, -183, -222, -423a, -450a, -450b, -451
**Controls**	*cel-miR-39-3p, **miRTC, ***PPC

*C. *elegans* miR-39

**reverse transcription and

***PCR controls

### Sample collection

Lymph node samples were collected by fine needle aspiration technique using a 22-gauge needle and 6mL syringe from one peripheral lymph node. Samples from lymphoma patients were collected at diagnosis and relapse by the Animal Cancer Centre staff at OVC and immediately placed into 200μL of RNA*later* stabilization solution (Life Technologies, Burlington, Canada) to be transported and then stored at -20°C. Samples from healthy control dogs were immediately placed into 700μL of QIAzol lysis reagent (Qiagen) and stored at -80°C. The presence of lymphocytes in control samples was confirmed by cytologic evaluation.

Blood samples were collected with a 22-gauge needle and 3 or 6mL syringe from the jugular or cephalic vein. Samples were immediately placed in an EDTA tube and processed within 24 hours. Whole blood was centrifuged at 609g for 10 minutes and then the supernatant (plasma) was removed and stored in 200μL aliquots at -80°C.

### RNA extraction

Lymph node samples stored in RNA*later* were diluted with 1000μL PBS buffer to decrease viscosity and then centrifuged at 5,000g for 5 minutes at room temperature to produce a cell pellet. The supernatant was then removed, and RNA extracted as directed by the manufacturer using the miRNeasy mini kit (Qiagen) starting with addition of 700μL QIAzol lysis reagent to the pellet. The control samples were previously stored in 700μL QIAzol lysis reagent. Homogenization of all samples was accomplished by vortexing. Then, 140μL of chloroform was added to the samples, mixed and centrifuged at 12,000g for 15 minutes at 4°C. The sample was then precipitated with 100% ethanol and additional Qiagen buffers in a column-based extraction method. RNA was eluted using 30μL of RNase-free water.

Frozen plasma samples were thawed, and RNA was extracted as directed by the manufacturer using the miRNeasy serum/plasma kit (Qiagen). QIAzol lysis reagent (1000μL) was added to a 200μL aliquot of plasma and homogenized by vortexing. An endogenous spike-in control (cel-miR-39) was added at this stage to monitor extraction efficiency for these samples. Next, 200μL of chloroform was added to the samples, mixed and centrifuged at 12,000g for 15 minutes at 4°C. The sample was then precipitated with 100% ethanol and additional Qiagen buffers in a column-based extraction method. RNA was eluted using 14μL of RNase-free water.

Total RNA concentration and contaminant identification (260/230 and 260/280 ratios) were assessed using a NanoDrop 2000 spectrophotometer (Thermo Fisher Scientific, Delaware, USA). miRNA concentration was measured using a Qubit 2.0 fluorometer (Life Technologies) and Qubit microRNA assay kit.

### Quantitative RT-PCR

Reverse transcription of RNA was performed using the miScript II RT kit as directed by the manufacturer (Qiagen). The reaction was prepared on ice using 50ng of template RNA from lymph node samples or 1.5μL of template RNA from plasma samples. The template RNA was added to the reverse-transcription master mix and incubated at 37°C for 60 minutes, followed by 95°C for 5 minutes. The cDNA samples were then diluted with 90μL of RNase-free water and stored at -20°C.

Quantitative reverse transcription polymerase chain reaction (RT-qPCR) was used to quantify the expression of 38 canine miRNAs. Eight samples at a time were run on a 384-well PCR array plate. For each sample, controls to assess RNA recovery (cel-miR-39), reverse transcription and PCR performance were included. The detection of cel-miR-39 was only assessed for plasma samples, which had the endogenous spike-in added during the RNA extraction process.

Using the miScript SYBR green PCR kit (Qiagen), 65μL of template cDNA was mixed with 265μL QuantiTect SYBR Green PCR master mix, 55μL miScript universal primer and 155μL RNase-free water. A Biomek NX robotic pipettor (Beckman Coulter, Mississauga, Canada) was used to add 10μL of the above solution to each well of the array plate containing primers. Subsequently, RT-qPCR was performed using a Roche LightCycler 480 II (Roche, Indianapolis, USA). The reaction was incubated at 95°C for 15 minutes, followed by 45 cycles of 94°C for 15 seconds, 55°C for 30 seconds and 70°C for 30 seconds.

### Data analysis

A parametric single-factor ANOVA was performed to assess differences in the range of age between the control group and lymphoma groups.

Quality control assays were analyzed for relative efficiency of reverse transcription and overall PCR performance using the GeneGlobe Data Analysis Center (Qiagen, www.qiagen.com). In addition, the plasma spike-in control was monitored to confirm extraction efficiency. NormFinder was utilized to identify two endogenous control miRNAs for normalization of RT-qPCR data and calculation of delta Ct values, which are inversely proportional to expression [13]. The target miRNA with the best stability value for each sample type (lymph node, plasma) in combination with one miRNA common to both sample types were chosen for this analysis. The Ct cut-off for lower limit of detection was set at 35. Relative quantification was determined using the ΔΔCt method [14]. Fold changes were calculated using 2^-(ΔΔCt)^ to assess for differences in miRNA expression between the control and diseased groups.

Differences in miRNA expression between healthy control dogs and patients with B and T cell lymphoma were determined. Furthermore, differences in expression between B and T immunophenotype were computed. These data were analyzed using a non-parametric Kruskal-Wallis one-way ANOVA with a Dunn’s multiple comparisons test.

Spearman correlation coefficients were used to assess correlation between lymph node aspirates and corresponding plasma samples. Samples were analyzed separately for each group: healthy control dogs, dogs with B cell lymphoma at diagnosis, dogs with B cell lymphoma at relapse and dogs with T cell lymphoma at diagnosis.

For analyzing miRNA profiles at diagnosis compared to relapse after CHOP chemotherapy, a Wilcoxon matched-pairs signed rank test was used with Spearman correlation for evaluating effective pairing for each target miRNA. For miRNAs that were significantly different at relapse, a Mann-Whitney test was used to compare expression at relapse to the healthy control dogs.

Differences in miRNA expression between clinical outcomes for patients receiving CHOP chemotherapy (remission vs non-remission, alive vs deceased at one year) were analyzed using a Mann-Whitney test. Lastly, Kaplan-Meier survival curves were established for PFS and OS using a log-rank (Mantel-Cox) test for miRNAs that were high-expressing or low-expressing based on median stratification of delta Ct values. Patients that were relapse free or alive at time of last contact were censored for analysis.

P-values less than 0.05 were considered statistically significant. All statistical analyses for the prospective study were performed using GraphPad Prism 7.0d (GraphPad Software, San Diego, CA, USA).

## Results

### Patient characteristics

Forty-five dogs in total were enrolled into the study to establish a control group and two test groups consisting of dogs with B and T cell lymphoma. These groups included 10 healthy control dogs, 22 dogs with B cell lymphoma and 13 dogs with T cell lymphoma. There was no statically significant difference in the range of age between these 3 groups (p-value = 0.1023). A summary of patient characteristics can be found in Table 2.

**Table 2:** Patient characteristics.

	Healthy controls (n = 10)	B cell lymphoma (n = 22)	T cell lymphoma (n = 13)
**Age range**	2–10 years (mean 5.6)	4–11 years (mean 7.3)	5–12 years (mean 7.7)
**Sex**	Male neutered n = 7 Female spayed n = 3	Male intact n = 2 Male neutered n = 13 Female spayed n = 7	Male neutered n = 7 Female spayed n = 6
**Breed**	Mixed breed n = 4 Australian shepherd n = 1 German shepherd n = 1 German shorthaired pointer n = 1 Labrador retriever n = 1 Rottweiler n = 1 Standard poodle n = 1	Mixed breed n = 7 Golden retriever n = 5 Labrador retriever n = 2 Border collie n = 1 Cocker spaniel n = 1 Doberman pinscher n = 1 German shepherd n = 1 Leonberger n = 1 Lhasa apso n = 1 Pembroke Welsh corgi n = 1 Viszla n = 1	Boxer n = 4 Golden retriever n = 2 Weimaraner n = 1 Mixed breed n = 1 Boston terrier n = 1 Dogue de Bordeaux n = 1 Jack Russell terrier n = 1 Labrador retriever n = 1 Pug n = 1

In addition to the forty-five dogs enrolled, a total of fourteen cases were excluded from this study. Nine were excluded due to concurrent diseases which included a diagnosis of additional neoplasms, uncontrolled endocrinopathy, renal disease and generalized demodicosis. Three dogs were excluded because they were being treated with prednisone and one dog could not be immunophenotyped. One dog met inclusion criteria but was later excluded because RNA could not be extracted from the lymph node sample.

### Quantification of miRNA in samples

Each sample was quantified using a Nanodrop for total RNA concentration followed by the Qubit fluorometer to confirm the presence of miRNA. A summary of total RNA and miRNA concentrations for lymph node aspirates and plasma samples can be found in S1 Fig.

### Raw Ct expression

For lymph node samples, all canine target miRNAs in the customized array were confirmed by successful amplification with all Ct values less than 35 in the control and lymphoma samples. For plasma samples, 32 out of 38 canine target miRNAs were successfully amplified in all samples with Ct values less than 35. Six miRNAs (miR-34a, miR- 127, miR-182, miR-183, miR-450a and miR-450b) had inconsistent amplification across plasma samples. The controls for RT efficiency and overall PCR performance passed for all samples. For all plasma samples, the spike-in control (cel-miR-39) was present with high amplification (Ct ≤26) confirming recovery after RNA extraction.

### Selection of endogenous controls for normalization

NormFinder ranked miR-16 for lymph node and miR-18a for plasma as the most stable candidate endogenous controls with stability values of 0.267 and 0.218, respectively. In addition, miR-27a was ranked amongst the top four targets common to both tissue types with stability values of 0.324 in lymph node and 0.248 in plasma. Therefore, the average of miR-16 and miR-27a were chosen to normalize lymph node data and the average of miR-18a and miR-27a for plasma data.

### Differential miRNA expression in lymphoma patients

Multiple miRNAs were found to have significantly different expression in dogs with B and T cell lymphoma compared to healthy control dogs. This included miRNAs that were common to both lymphoma groups and others that were immunophenotype-specific.

#### Lymph node samples

**Fig 1:**
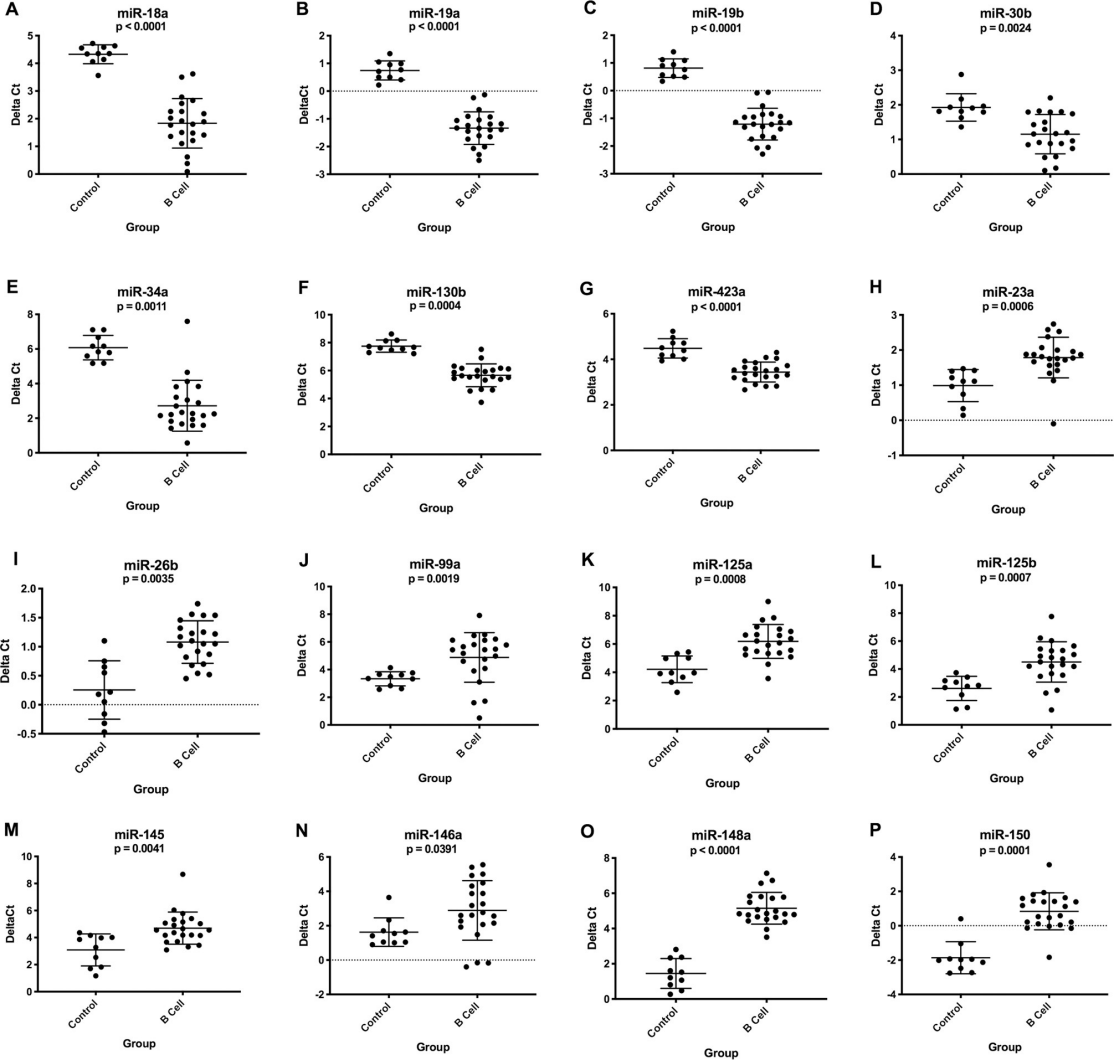
Graphs showing miRNA expression (delta Ct) in lymph node aspirates from healthy control dogs (n = 10) compared to B cell lymphoma patients at diagnosis (n = 22). These miRNAs had either significantly upregulated (A-G) or downregulated (F-P) expression in the B cell lymphoma group. (Kruskal-Wallis one-way ANOVA with Dunn’s multiple comparisons test, p-value <0.05; additional group comparisons are shown in Fig 2 and S2 Fig). Error bars represent mean +/- standard deviation.

**Fig 2:**
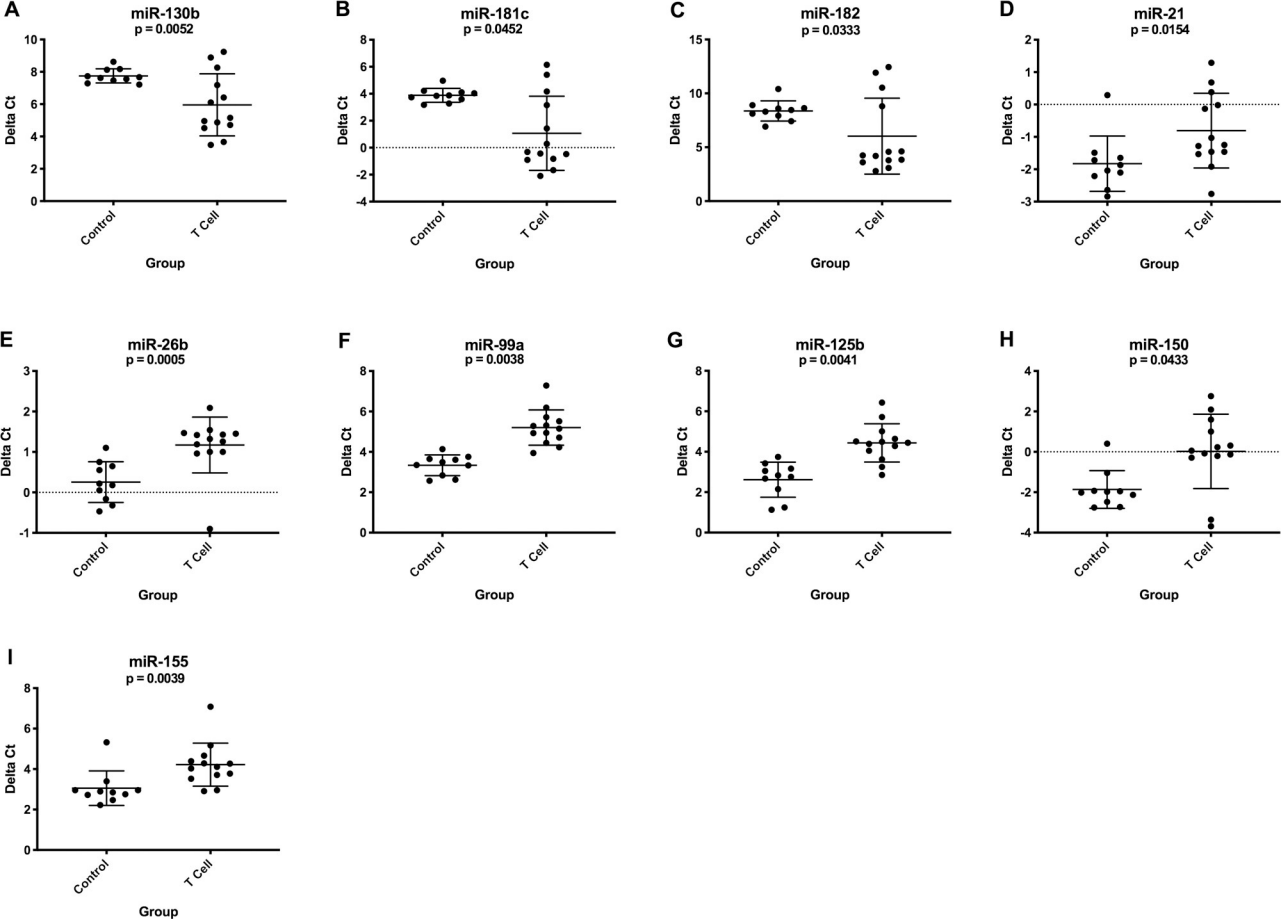
Graphs showing miRNA expression (delta Ct) in lymph node aspirates from healthy control dogs (n = 10) compared to T cell lymphoma patients at diagnosis (n = 13). These miRNAs had either significantly upregulated (A-C) or downregulated (D-I) expression in the T cell lymphoma group. (Kruskal-Wallis one-way ANOVA with Dunn’s multiple comparisons test, p-value <0.05; additional group comparisons are shown in Fig 1 and S2 Fig). Error bars represent mean +/- standard deviation.

For B cell lymphoma, 7 miRNAs were upregulated and 9 were downregulated in lymph node samples compared to healthy controls (Fig 1). The miR-19a/19b cluster (Fig 1B and 1C) and miR-148a (Fig 1O) had no overlap in expression between the lymphoma group and control dogs. For T cell lymphoma, 3 miRNAs were upregulated and 6 were downregulated in lymph nodes compared to healthy controls (Fig 2). Both immunophenotypes had upregulation of miR-130b and downregulation of miR-26b, miR-99a, miR-125b and miR-150. The expression levels of 17 miRNAs were significantly different when compared between immunophenotypes (S2 Fig). S1 and S2 Tables list average delta Ct values, fold changes and p-values.

#### Plasma samples

**Fig 3:**
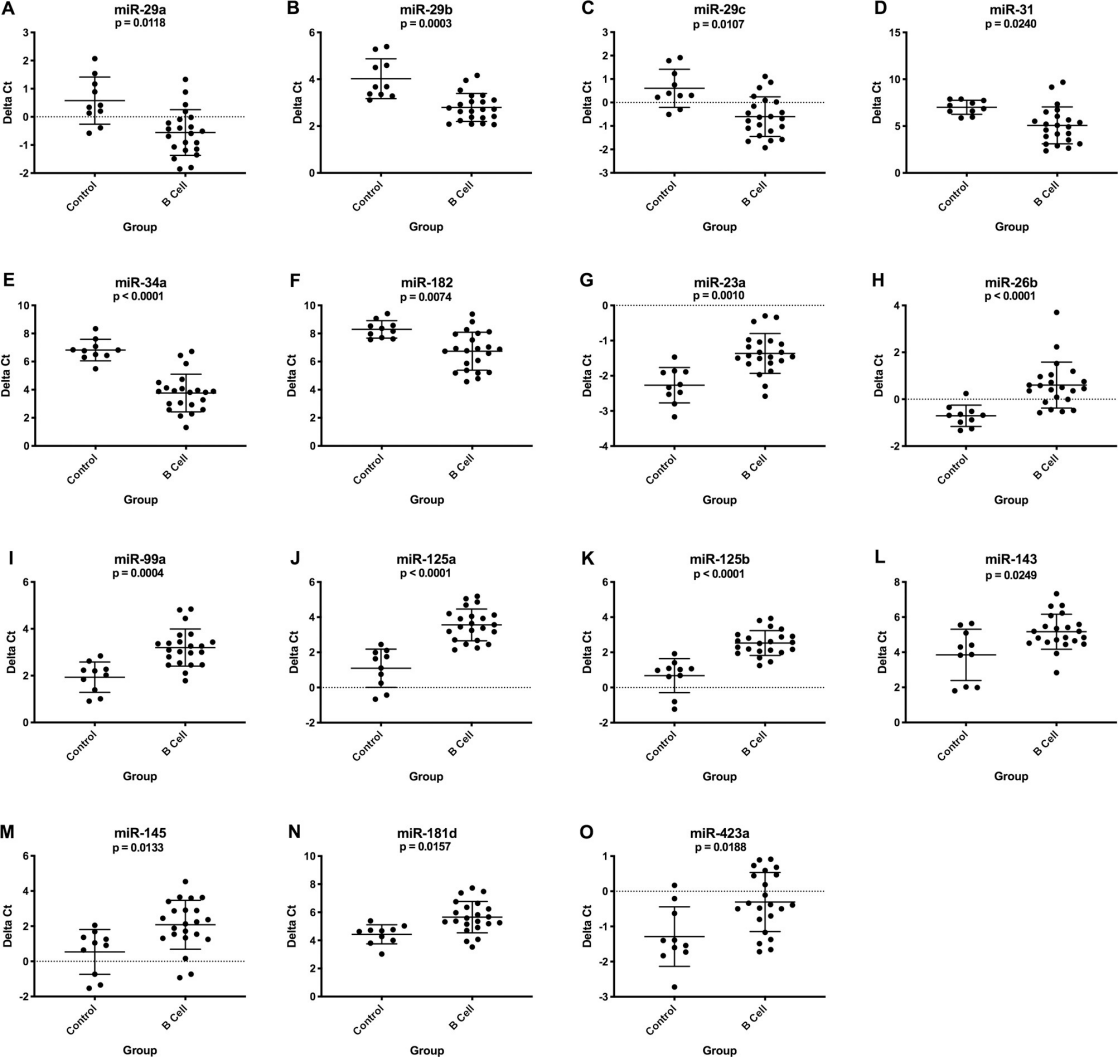
Graphs showing miRNA expression (delta Ct) in plasma from healthy control dogs (n = 10) compared to B cell lymphoma patients at diagnosis (n = 22). These miRNAs had either significantly upregulated (A-F) or downregulated (G-O) expression in the B cell lymphoma group. (Kruskal-Wallis one-way ANOVA with Dunn’s multiple comparisons test, p-value <0.05; additional group comparisons are shown in Fig 4 and S3 Fig). Error bars represent mean +/- standard deviation.

**Fig 4:**
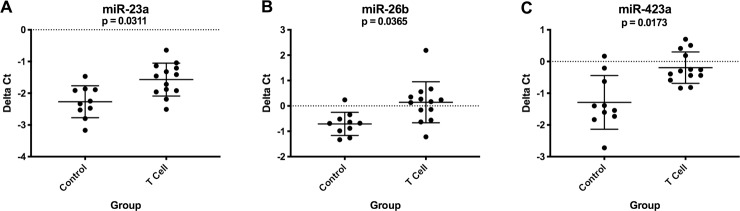
Graphs showing miRNA expression (delta Ct) in plasma from healthy control dogs (n = 10) compared to T cell lymphoma patients at diagnosis (n = 13). These miRNAs had significantly downregulated (A-C) expression in the T cell lymphoma group. (Kruskal-Wallis one-way ANOVA with Dunn’s multiple comparisons test, p-value <0.05; additional group comparisons are shown in Fig 3 and S3 Fig). Error bars represent mean +/- standard deviation.

For B cell lymphoma, 6 miRNAs were upregulated and 9 were downregulated in plasma samples compared to healthy controls (Fig 3). For T cell lymphoma, 3 miRNAs were downregulated in plasma compared to healthy controls (Fig 4). Both immunophenotypes had downregulation of miR-23a and miR-26b. The expression levels of 14 miRNAs were significantly different when compared between immunophenotypes (S3 Fig). S3 and S4 Tables list average delta Ct values, fold changes and p-values.

Similar alterations for miR-34a, miR-23a, miR-26b, miR-99a, miR-125a, miR-125b and miR-145 in B cell lymphoma and miR-26b in T cell lymphoma were present in both the lymph node aspirates and plasma samples.

### Correlation of expression between lymph node and plasma samples

The majority of miRNAs had different expression levels between lymph node aspirates and corresponding plasma samples. Expression of a single miRNA, miR-143, was significantly correlated between lymph node and plasma in the healthy control dogs. In the B cell lymphoma group, 6 miRNAs had correlated expression between lymph node and plasma at diagnosis and 2 different miRNAs were correlated at relapse. In the T cell lymphoma group, 8 miRNAs had correlated expression between lymph node and plasma at diagnosis. The strength of the correlations ranged from moderate to very strong [15]. miR-130b and miR-181b had correlated expression in both the B and T cell lymphoma groups at diagnosis. miR-99a in the B cell lymphoma group at diagnosis was the only miRNA that was previously found to have significantly altered expression in both sample types when compared to healthy control dogs. Table 3 summarizes the miRNAs with significant correlation of expression, their correlation coefficients (Spearman rho) and p-values.

**Table 3:** miRNAs with significant correlation of expression levels between lymph node aspirates and plasma samples from the healthy controls, B cell lymphoma group at diagnosis and relapse and T cell lymphoma group at diagnosis (Spearman correlation, p value <0.05).

Group	Target miR	rho	P-value
**Healthy controls**	cfa-miR-143	-0.6727	0.0390
**B cell @ diagnosis**	cfa-miR-181b	0.6441	0.0012
	cfa-miR-181a	0.6149	0.0023
	cfa-miR-31	0.6141	0.0024
	cfa-miR-99a	0.5847	0.0043
	cfa-miR-146a	0.5264	0.0118
	cfa-miR-30b	0.4440	0.0384
	cfa-miR-130b	0.4429	0.0390
**B cell @ relapse**	cfa-miR-21	0.9286	0.0067
	cfa-miR-23a	-0.7857	0.0480
**T cell @ diagnosis **	cfa-miR-182	0.8190	0.0010
	cfa-miR-143	-0.7680	0.0030
	cfa-miR-130b	0.6870	0.0120
	cfa-miR-183	0.6870	0.0120
	cfa-miR-450a	0.6810	0.0130
	cfa-miR-450b	0.6700	0.0150
	cfa-miR-181b	0.6210	0.0270
	cfa-miR-23a	0.5660	0.0470

### Differential miRNA expression at relapse compared to diagnosis

**Fig 5:**
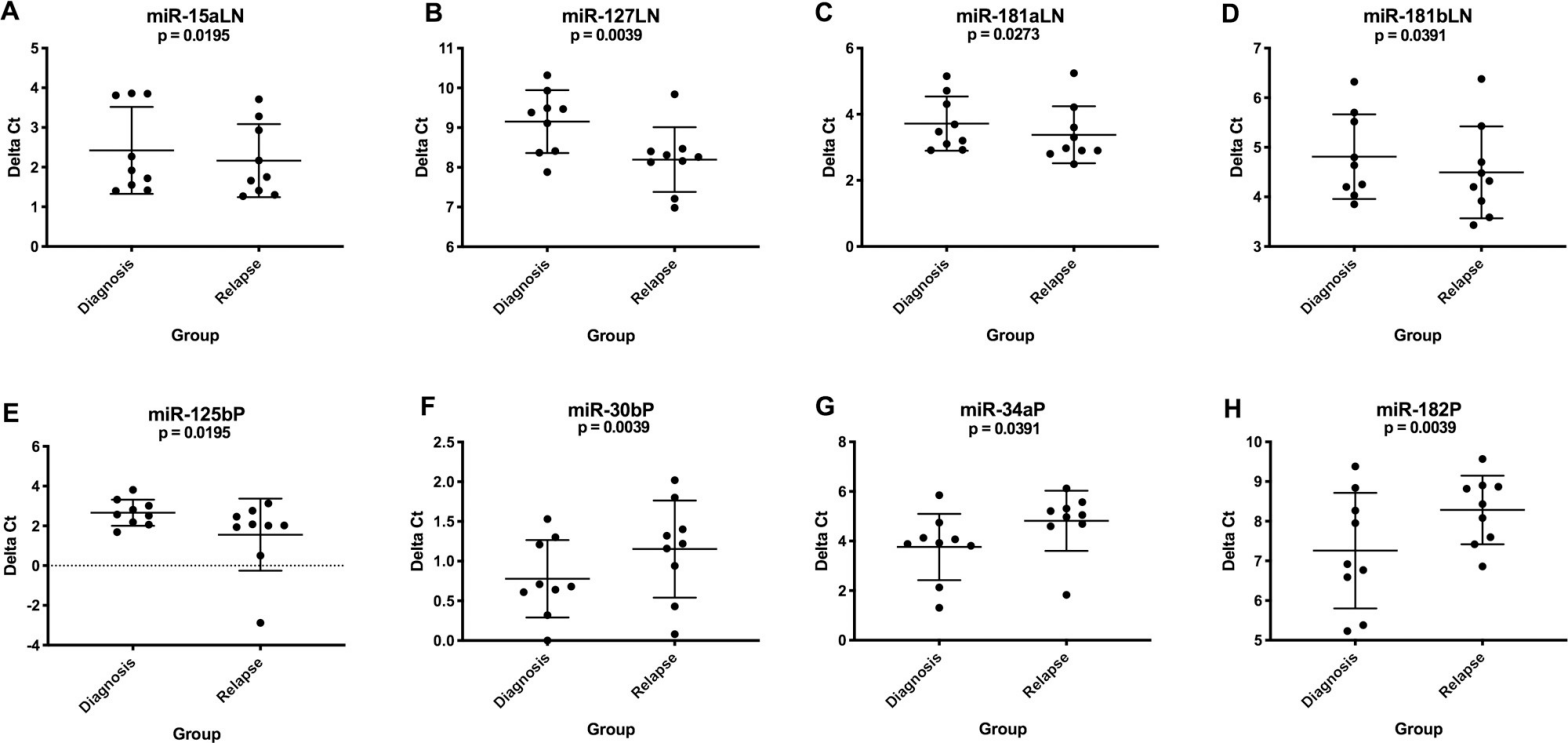
Graphs showing miRNA expression (delta Ct) in B cell lymphoma patients at diagnosis (n = 9) and relapse (n = 9). These miRNAs had significantly altered expression in lymph nodes (A-D) or plasma (E-H) at relapse compared to expression at time of diagnosis. (Wilcoxon matched-pairs signed rank test, two-tailed p-value <0.05). LN = lymph node, P = plasma.

Nine dogs with B cell lymphoma and paired lymph node and plasma samples at diagnosis and relapse were included in this analysis. Two dogs relapsed during CHOP chemotherapy and seven relapsed after completion of therapy. Relapse was recognized as recurrence of peripheral lymphadenopathy. Seven cases of relapse were confirmed by cytology and one by post-mortem histopathology. There were 4 miRNAs with significant upregulation in lymph node aspirates from dogs with B cell lymphoma at relapse compared to expression at diagnosis (Fig 5A–5D). In plasma, there was a single miRNA with significant upregulation (Fig 5E) and 3 with downregulation (Fig 5F–5H). S5 Table lists average delta Ct values, fold changes and p-values.

For miRNAs that were significantly different at relapse compared to diagnosis, miR-127 (p-value = 0.0006) in lymph node and miR-34a (p-value <0.0001) and miR-125b (p-value = 0.0279) in plasma were the only miRNAs with altered expression at relapse when compared to the healthy control dogs.

### Relationship between miRNA expression and clinical outcome

**Fig 6:**
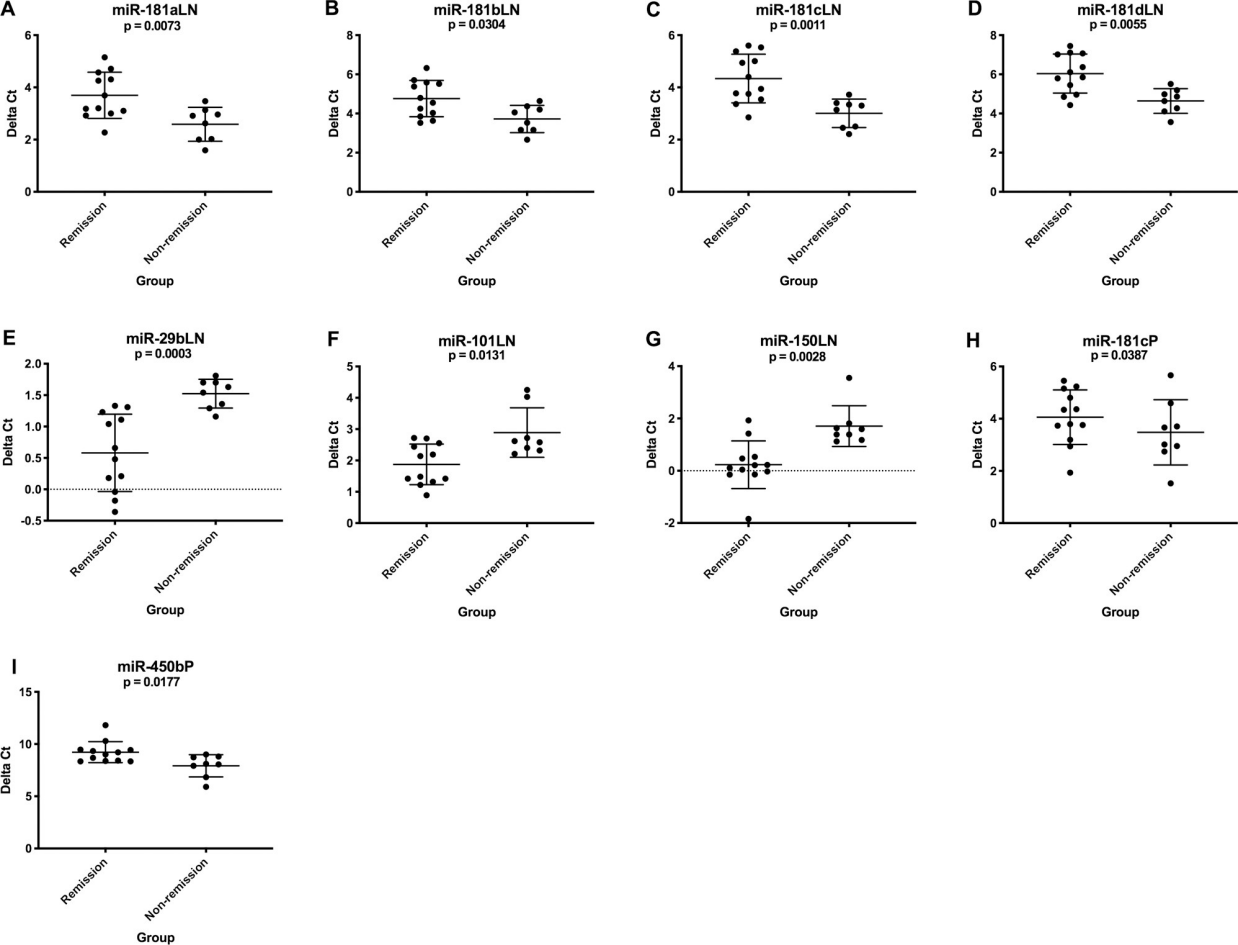
Graphs showing miRNA expression (delta Ct) at diagnosis in B cell lymphoma patients that completed CHOP in complete remission (n = 12) versus non-remission (n = 8). These miRNAs had significantly altered expression associated with response to therapy. (Mann-Whitney test, two-tailed p-value <0.05). LN = lymph node, P = plasma. Error bars represent mean +/- standard deviation.

**Fig 7:**
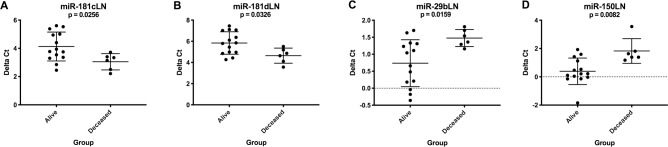
Graphs showing miRNA expression (delta Ct) at diagnosis in B cell lymphoma patients that were alive at one year (n = 14) versus deceased (n = 6). These miRNAs had significantly altered expression associated with one-year survival. (Mann-Whitney test, two-tailed p-value <0.05). LN = lymph node, P = plasma. Error bars represent mean +/- standard deviation.

Several analyses were performed to identify miRNAs associated with prognosis for patients receiving CHOP chemotherapy. Only dogs with B cell lymphoma were assessed in the first two analyses because of limited sample sizes for T cell lymphoma. Complete remission was defined as regression of measurably palpable lymph nodes to non-pathologic size based on Veterinary Cooperative Oncology Group response evaluation criteria [16]. Eight dogs were included in the non-remission group (which included both non-responders and dogs that relapsed during CHOP) and twelve dogs completed CHOP in complete remission. There was significant upregulation in expression of the miR-181 family and downregulation of miR-29b, miR-101 and miR-150 in lymph nodes at diagnosis for the non-remission group compared to dogs that completed CHOP in complete remission (Fig 6A–6G). In plasma, miR-181c and miR-450b had significantly higher expression at diagnosis in the non-remission group (Fig 6H and 6I). Fourteen dogs were alive and six were deceased at one year after diagnosis. For one-year survival, dogs with lymphoma that died prior to one year had higher expression of the miR-181c/181d cluster and lower expression of miR-29b and miR-150 in lymph nodes at diagnosis (Fig 7). No significant differences in one-year survival were observed for plasma miRNAs. S6 and S7 Tables list average delta Ct values, fold changes and p-values.

**Fig 8:**
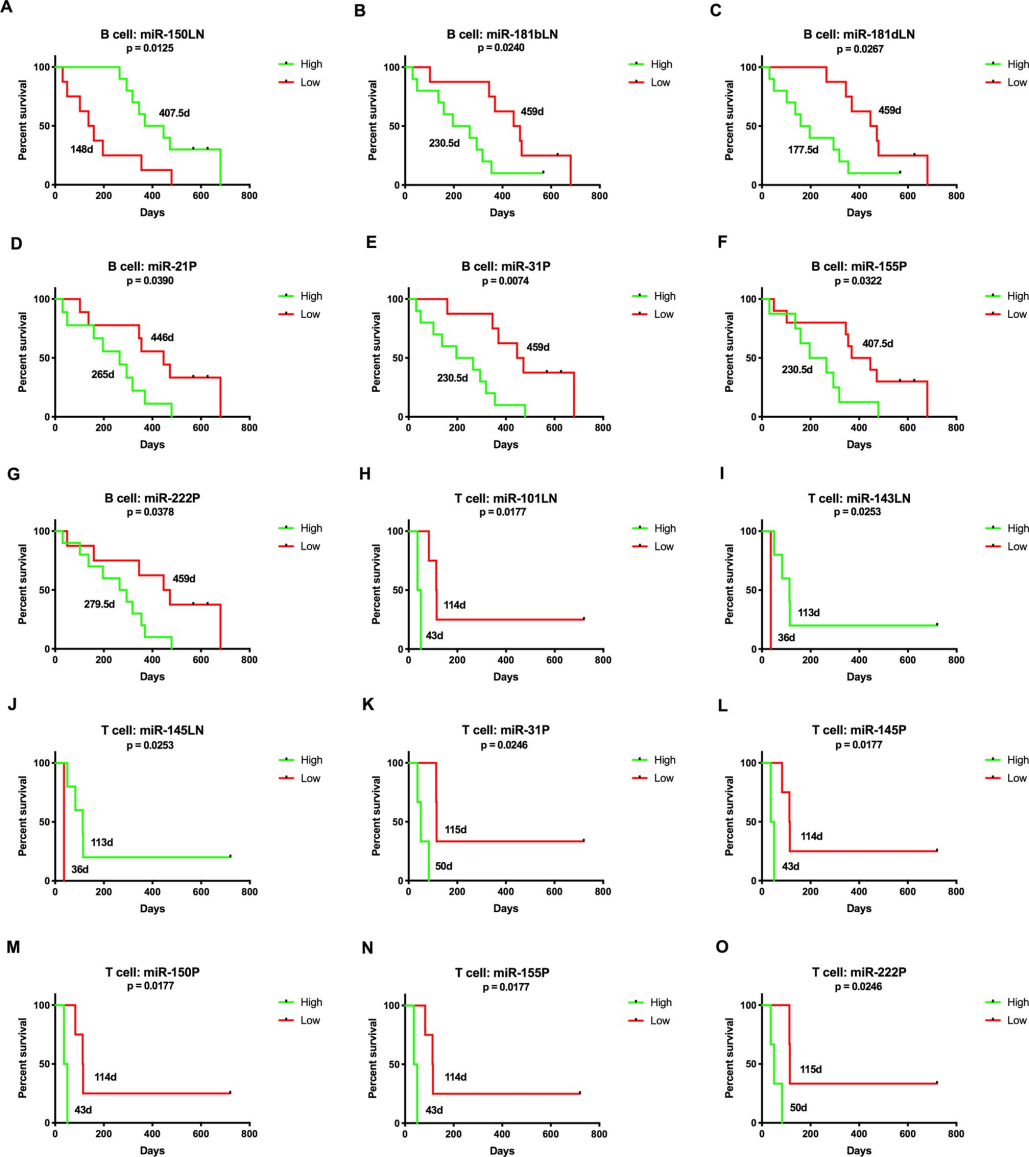
Kaplan-Meier curves for progression-free survival comparing high and low miRNA expression at diagnosis for B (n = 18) and T (n = 6) cell lymphoma. These miRNAs had expression levels significantly correlated with progression-free survival. Values on the curve represent median survival times. (Log-rank (Mantel-Cox) test, p-value <0.05).

**Fig 9:**
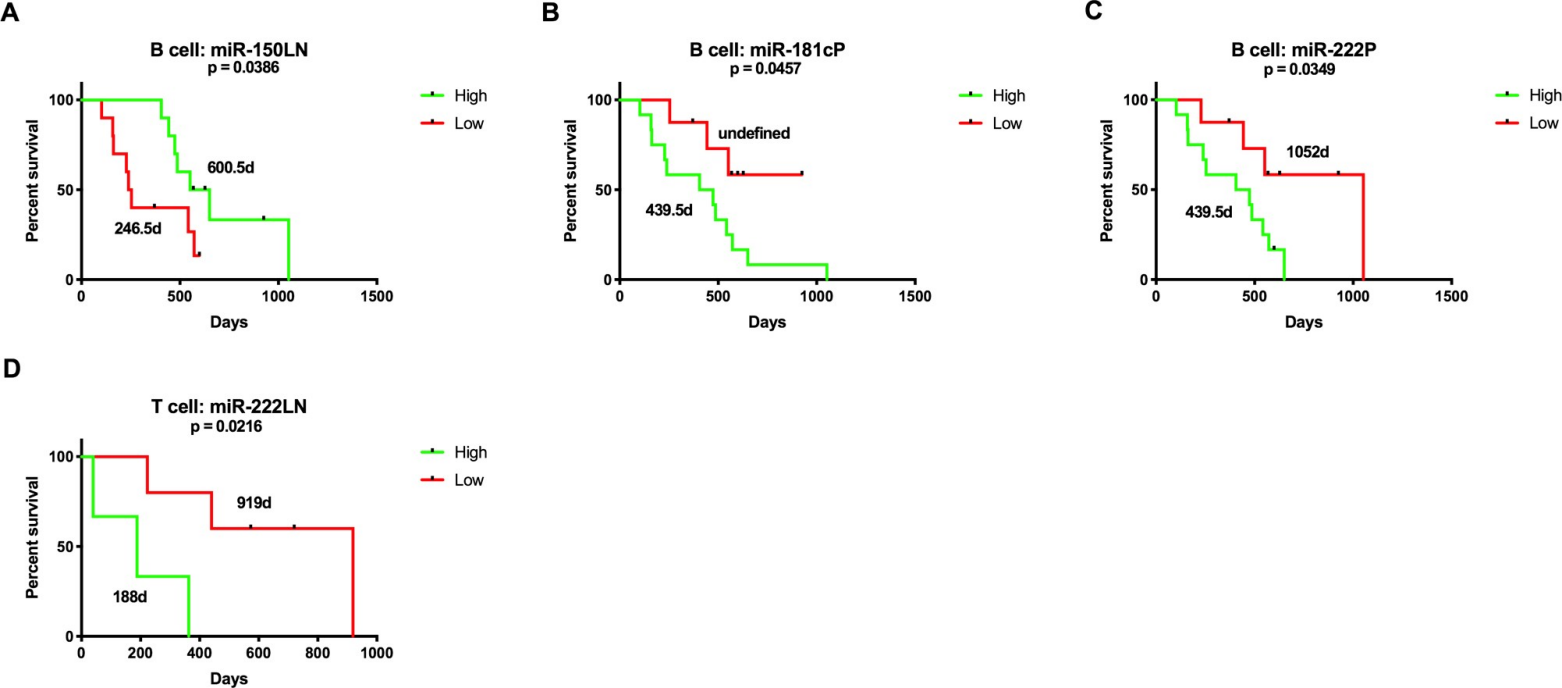
Kaplan-Meier curves for overall survival comparing high and low miRNA expression at diagnosis for B (n = 20) and T (n = 8) cell lymphoma. These miRNAs had expression levels significantly correlated with overall survival. Values on the curve represent median survival times. (Log-rank (Mantel-Cox) test, p-value <0.05).

Association of high versus low miRNA expression was significantly correlated with PFS and OS for several miRNAs. For B cell lymphoma, high expression of miR-150 in lymph nodes was positively correlated (Figs 8A and 9A) and miR-222 in plasma was negatively correlated (Figs 8G and 9C) with both PFS and OS. In addition, expression of the miR-181 family, miR-21, miR-31 and miR-155 were negatively correlated with survival (Figs 8 and 9). For T cell lymphoma, miR-31, miR-101, miR-150, miR-155 and miR-222 expression were negatively correlated with survival (Figs 8 and 9). Expression of the miR-143/145 cluster was positively correlated with PFS for lymph node samples (Fig 8I and 8J), but miR-145 was negatively correlated for plasma (Fig 8L). S8 and S9 Tables list median survival times and p-values.

## Discussion

Canine lymphoma is a spontaneous neoplasm that shares many features with the human disease [1]. It has been proposed that the dog is a valuable animal model for human cancer research as they share the same environment and are exposed to similar factors that may promote cancer development [16]. The discovery of miRNAs as effective biological markers for human cancer is an active area of research, however, there is currently limited studies exploring miRNAs in canine neoplasms. The results of this study highlight multiple miRNAs that may be of interest for detection and prognostication of canine lymphoma.

Normalization of data prior to interpretation is an important step for differentiating experimentally induced variation from true biological change [17]. We opted to use the NormFinder algorithm to identify suitable endogenous controls by ranking miRNAs according to their expression stability [13]. Notably, miR-16 was determined to be the most stable endogenous control for lymph node tissue in our study, which is consistent with previous canine studies [8, 9].

The first objective of this study was to identify miRNAs in lymph nodes and plasma that had differential expression in dogs with lymphoma compared to healthy control dogs. Many miRNAs have been documented in the human literature to play functional roles in lymphocyte development and lymphomagenesis. We observed altered expression in several of these miRNAs in canine lymphoma patients. In addition, some of these alterations have previously been observed in other canine lymphoma studies [7, 8]. miRNAs associated with carcinogenesis are often presumed to act as “oncomiRs” if upregulated or tumour suppressor miRNAs if downregulated, though many have dual function depending on cancer type [5]. This duality is thought to result from different cell types having unique expression profiles and a diversity of targets that may be repressed [18].

The miR-17-92 cluster, miR-155, miR-21 and miR-125b are confirmed oncomiRs in human lymphoma [19]. The miR-17-92 cluster is important in early B cell maturation and is often amplified in human hematopoietic malignancies including DLBCL [20]. miR-18a, miR-19a and miR-19b are part of this cluster and were significantly upregulated in lymph nodes from dogs with B cell lymphoma in our study. In transgenic mice, overexpression of this cluster leads to rapid lymphoproliferation by promoting B cell progenitor survival through repression of pro-apoptotic BCL2 like 11 (*BCL2L11/BIM*) and phosphatase and tensin homolog (*PTEN*) [21]. Uhl et al (2011) also found these miRNAs to be upregulated in canine B cell lymphoma [7].

miR-155 is highly expressed in murine activated B and T cells to regulate homeostasis and immune function [22]. In human T cell development, miR-21 aids in activation and proliferation by targeting the tumor suppressor programmed cell death 4 (*PDCD4*) [23]. Aberrant overexpression of miR-155 and miR-21 has been observed in natural killer/T cell lymphoma with concurrent downregulation of multiple tumour suppressors resulting in activation of AKT signaling [24]. Conversely, we found miR-155 and miR-21 to be downregulated in lymph nodes from dogs with T cell lymphoma suggesting these miRNAs may act as tumour suppressors rather than oncomiRs in canine lymphoma.

Overexpression of miR-125b has been associated with chromosomal abnormalities in human lymphoid malignancies and ectopic expression in hematopoietic stem cells can lead to lymphoproliferative disorders [25]. This miRNA has an anti-apoptotic effect on murine stem cells and can promote lymphoid-fate decisions [26]. Moreover, miR-125b regulates human B and T cell maturation by downregulating key transcription factors required for differentiation [27, 28]. In our lymphoma groups, there was a contradictory downregulation of the miR-125a/125b cluster in lymph node and plasma. Perhaps this alteration contributes to canine lymphomagenesis due to loss of control over lymphocyte differentiation.

In contrast, miR-34a and miR-150 are generally considered tumour suppressor miRNAs in human lymphoma. miR-34a is often lost or epigenetically silenced in many human cancers [29, 30]. In murine bone marrow this miRNA has been shown to inhibit the expression of forkhead box P1 (*FOXP1*), which is involved in the pro-B to pre-B transition during normal lymphocyte development and is considered an oncogene in mature B cells [31]. Therefore, overexpression in lymph node and plasma for our B cell lymphoma patients was an unexpected finding. This miRNA may alternatively act as an oncomiR in canine lymphoma or may be increased as a consequence to positive feedback from the p53 pathway, which can be stimulated by cellular stress responses [32, 33].

miR-150 acts as a tumor suppressor that is consistently downregulated in the majority of human lymphomas [34]. Roehle et al (2008) reported that miR-150 was the most strongly downregulated miRNA in DLBCL tissue compared to normal tissue [35]. Downregulation of miR-150 in canine lymphoma patients is therefore compatible with the human literature. This miRNA is selectively expressed in murine mature resting B and T lymphocytes and therefore may also reflect a decrease in the proportion of resting lymphocytes in the neoplastic lymph nodes.

Overexpression of the miR-181a/181b cluster and miR-183 have previously been identified in canine multicentric T cell lymphoma [7, 8]. In the current study, upregulation of miR-181c was observed in lymph nodes from dogs with T cell lymphoma. The miR-181 family is essential for both B and T cell development and participates in thymic differentiation by sensitizing double positive cells to antigen responses aiding in positive and negative selection [36]. Upregulation of miR-182, which is clustered with miR-183, was found in lymph nodes from dogs with T cell lymphoma, which supports an oncogenic role for these miRNAs in canine T cell lymphoma.

While we found differential miRNA expression in canine lymphoma patients compared to normal dogs, we cannot be certain if these alterations contribute to pathogenesis. In the lymph nodes, altered expression may reflect biological effects or may simply be a consequence of the lymph node becoming filled with a clonal population that is essentially one subtype of lymphocyte that also happens to be neoplastic. Furthermore, the origin of miRNA released into circulation is often unknown and could arise from multiple tissues. We cannot conclude if these miRNAs are associated with oncogenic or tumour suppressive effects on the neoplastic cells or if they are released in response to the neoplasm.

The next objective in our study was to compare miRNA profiles from lymph nodes to corresponding plasma samples in dogs with lymphoma and healthy controls. The ability to identify meaningful biomarkers in plasma would be ideal as blood is a routinely collected sample type and more conveniently processed for shipping and storage. Only a single miRNA was correlated between lymph node and plasma in the healthy control group, but several miRNAs had significant correlation in the lymphoma groups. This difference suggests there may be greater production and release of these miRNAs from neoplastic lymphocytes resulting in plasma levels that more closely mirrored these alterations. miR-99a in the B cell lymphoma group was the only miRNA with differential expression identified in both lymph node and plasma at diagnosis and showed moderate correlation in expression levels between the sample types. Based on these findings, miR-99a may have potential diagnostic use as a circulating biomarker in plasma that reflects alterations in lymph node. This miRNA is thought to act as a tumour suppressor in human DLBCL and was significantly downregulated in our canine patients [35].

Lymph node and plasma samples obtained from B cell lymphoma patients at relapse were profiled and compared to expression levels at time of diagnosis. The goal was to identify changes in expression that may be associated with disease aggressiveness or chemoresistance, which may serve as potential therapeutic targets. Relapse usually represents the emergence of tumour cells that are more resistant to chemotherapy than the original tumour [37]. Surprisingly, in this study only eight miRNAs showed significantly different expression at relapse.

miR-125b in plasma was downregulated in dogs with B cell lymphoma compared to healthy control dogs, but within the B cell lymphoma group it was significantly higher at relapse compared to expression at diagnosis. Overexpression of this miRNA has been shown to directly target TNF alpha induced protein 3 (*TNFAIP3*) to activate the NF-κB pathway which is associated with aggressiveness in human DLBCL cell lines [38]. Furthermore, monitoring levels of circulating miR-125b in DLBCL patients has demonstrated its involvement in recurrence, progression and chemoresistance [39]. In addition, miR-34a and miR-182 were upregulated in plasma at diagnosis compared to healthy control dogs, but these levels decreased at relapse. It is unclear if these miRNAs contribute to increased aggressiveness or if they reflect chemotherapy-induced normalization of miRNA dysregulation since expression at relapse more closely resembled expression of the healthy control group.

miR-127 was upregulated at relapse in lymph node and was only significantly different from healthy control dogs at this time point. This miRNA may be associated with chemoresistance and, if proven, miR-127 could be considered a potential therapeutic target for sensitization. On the other hand, miR-127 upregulation may be a consequence of chemotherapy. This miRNA has been shown to target the proto-oncogene *BCL6* transcription repressor and therefore increased expression may suggest heightened tumour suppressor activity after chemotherapy. This phenomenon has been observed with chromatin-modifying drugs that activate expression of miR-127 in human cancer cells [40].

Lastly, we sought to identify miRNAs associated with outcome for canine lymphoma patients treated with CHOP chemotherapy. Prediction of patient response to therapy may provide a valuable tool for clinician and client decision making.

Higher expression of the miR-181 family members at diagnosis was associated with inferior outcome in the B cell lymphoma group. In contrast, higher expression of miR-181a in human DLBCL is associated with improved PFS in patients treated with R-CHOP and it has been shown to target the oncogene *FOXP1* in cell lines [41]. Conversely, lower expression of miR-150 and miR-29b was negatively associated with outcome which is consistent with the human literature. A recent study demonstrated that MYC-induced downregulation of miR-150 in human follicular lymphoma tissue contributed to high-grade transformation and shorter OS [42]. Evidence suggests that miR-29b plays a vital role in chemoresistance and pretreatment expression can be predictive of response in several human cancer types. It has been shown to improve chemosensitivity through targeting oncogenes, epigenetic modification and apoptosis [43].

In B and T cell lymphoma patients, we found that higher expression of miR-222 at diagnosis was associated with shorter PFS and OS. The COO signature and prognostic panels for human DLBCL described by Montes-Morena et al (2011) both include this miRNA [6]. For both lymphoma groups higher expression levels of miR-155 in plasma at diagnosis were associated with shorter PFS. In human DLBCL, Zhong et al (2012) observed that patients with higher expression of miR-155 in tissue achieved lower complete remission and response rates and shorter PFS [44]. In our study, only expression in plasma was correlated with survival and therefore the origin of production and release or effect on lymphoid tissue is unknown.

Higher expression of miR-21 in plasma at diagnosis from dogs with B cell lymphoma was negatively correlated with PFS. This miRNA was one of the earliest identified “oncomiRs” targeting numerous tumor suppressor genes associated with human cancers [45]. It has been observed to decrease sensitivity of DLBCL cells to CHOP chemotherapy by targeting *PTEN*, which negatively regulates the PI3K/AKT pathway [46].

Sample size was a limitation in this study, particularly for recruitment of patients with T cell lymphoma. Moreover, T cell lymphoma is a more heterogeneous disease than B cell lymphoma which is dominated by the DLBCL type [47,48]. A subset of the T cell lymphoma patients was concurrently enrolled in another study which meant for seven dogs we received a histomorphologic diagnosis. There were three peripheral T cell lymphomas—not otherwise specified, two T lymphoblastic lymphomas and two T zone lymphomas. We were not able to consider these subtypes separately in our study due to limited numbers. Ideally, future studies should strive to investigate T cell lymphoma subtypes as separate entities especially since we know indolent forms exist. In addition, our study only focused on samples from lymphoma patients in comparison to healthy control dogs. Establishing expression profiles for non-neoplastic diseases causing lymphadenopathy would further clarify the specificity of cancer associated miRNAs. Inclusion of alternative etiologies such as follicular hyperplasia from antigenic stimulation and lymphadenitis from infectious processes should be considered in follow up studies.

Interference caused by hemolysis was not investigated for plasma or lymph node samples. Hemolysis can result in significant variation of miRNAs highly expressed in erythrocytes due to release from ruptured cells. In the human literature, Blondal et al (2013) established a hemolysis indicator based on the level of erythrocyte-specific miR-451 in relation to miR-23a, which is a relatively stable miRNA in human plasma [49]. It was concluded that a delta Ct (miR-23a minus miR-451) greater than 7 indicates a high risk of hemolysis affecting the data obtained. We included these miRNAs in our custom PCR array, however it was later decided not to disqualify samples based on this guideline since its relevance to canine plasma is currently unknown. Establishing a cut-off value for hemolytic interference based spectrophotometric hemoglobin concentrations may be helpful for subsequent studies and allow exclusion of samples prior to further processing. Not all miRNAs are affected by hemolysis but applying indicator cut-offs would improve specificity of miRNA signatures [49].

miRNAs are known for their robust stability, even in body fluids [5]. Individual miRNAs have demonstrated differential rates of degradation with certain transcripts more susceptible than others. In circulation, cell-free miRNAs associated with lipid-based vesicles are more resistant to RNase degradation [50]. Quality control for RNA integrity was not performed in this study. Assessment can be important for reproducibility and accuracy in profiling studies, although it has been shown that miRNAs can remain stable even in degraded RNA preparations [51]. We opted to quantify total RNA using a Nanodrop, which only provides concentration and purity measurements. An additional step to determine total RNA quality (RIN = RNA integrity number) may be implemented in future studies. Samples were also evaluated with the Qubit miRNA assay. However, the resulting average fractions represented approximately 20% of total RNA in tissue and 5% in plasma suggesting this assay is not specific to small RNA and other RNA species may be detected.

Many of the target miRNAs we identified have been previously characterized in human lymphoma supporting the concept that miRNAs are highly conserved between species with possible functional relationships leading to similar disease processes [3, 52]. Target prediction and functional studies for miRNAs of interest were beyond the scope of this study. Therefore, we are not able to conclude how differential expression observed in these patients contributes to the biology and behaviour of lymphomagenesis. Establishing similarities in lymphoma-associated miRNA expression and gene targeting between canine and human lymphoma would provide additional evidence that spontaneously arising lymphoma in the dog is an appropriate animal model for human lymphoma research.

## Conclusions

This study highlights miRNAs of interest that were associated with immunophenotype and clinical outcome for canine multicentric lymphoma.

We identified novel miRNA alterations in addition to findings described in other previous canine studies. Notably, upregulation of the miR-17-92 cluster, the miR-29 family and miR-34a were strongly associated with B cell lymphoma and upregulation of the miR-181 family with T cell lymphoma. These miRNAs may be useful for lymphoma diagnosis and predicting immunophenotype from clinical lymph node samples. There was no overlap in lymph node expression between the healthy dogs and those with B cell lymphoma for miR-19a, miR-19b and miR-148, suggesting these miRNAs could provide good predictive value as biomarkers.

There were very few miRNAs with altered expression at relapse compared to diagnosis. Upregulation of miR-127 in lymph nodes was only observed in dogs with B cell lymphoma compared to healthy control dogs at time of relapse and therefore may be associated with chemoresistance or chemotherapy-induction.

Our final objective was to identify miRNAs associated with outcome for canine lymphoma patients treated with CHOP chemotherapy. For B cell lymphoma, upregulation of the miR-181 family and downregulation of miR-29b and miR-150 were associated with inferior response to therapy and survival. Higher expression of miR-155 and miR-222 were negatively associated with outcome in both B and T cell lymphoma.

Future goals include development of miRNA signatures that may be effective as minimally invasive biomarkers. Additionally, target identification and exploring gene-miRNA-protein networks would contribute to a better understanding of pathogenic roles and insight for the development of targeted therapies. This study provides a framework for future molecular and functional investigations with the ultimate goal of providing more personalized medicine to veterinary cancer patients.

## Supporting information

S1 Fig Graphs showing total RNA and miRNA concentrations (ng/μL) in lymph node aspirates and plasma samples from healthy control dogs and patients with B and T cell lymphoma. Error bars represent mean +/- standard deviation.
(TIF)

S2 Fig Graphs showing miRNA expression (delta Ct) from lymph node aspirates in B cell lymphoma (n = 22) and T cell lymphoma patients (n = 13). These miRNAs had significantly higher expression in B cell (A-L) or T cell (M-Q) lymphoma compared to the other immunophenotype. (Kruskal-Wallis one-way ANOVA with Dunn’s multiple comparisons test, p-value <0.05; additional group comparisons are shown in Figs 1 and 3). Error bars represent mean +/- standard deviation.
(TIF)

S3 Fig Graphs showing miRNA expression (delta Ct) from plasma in B cell lymphoma (n = 22) and T cell lymphoma patients (n = 13). These miRNAs had significantly higher expression in B cell (A-G) or T cell (H-N) lymphoma compared to the other immunophenotype. (Kruskal-Wallis one-way ANOVA with Dunn’s multiple comparisons test, p-value <0.05; additional group comparisons are shown in Figs 3 and 5). Error bars represent mean +/- standard deviation.
(TIF)

S1 Table miRNAs with significant change in expression in lymph nodes for dogs with B and T cell lymphoma at diagnosis compared to healthy controls. (DOCX)

S2 Table miRNAs with significantly higher expression in lymph nodes at diagnosis for dogs with B or T cell lymphoma compared to the other immunophenotype. (DOCX)

S3 Table miRNAs with significant change in expression in plasma for dogs with B and T cell lymphoma at diagnosis compared to healthy controls. (DOCX)

S4 Table miRNAs with significantly higher expression in plasma at diagnosis for dogs with B or T cell lymphoma compared to the other immunophenotype. (DOCX)

S5 Table miRNAs with significant change in expression at relapse compared to expression at time of diagnosis for dogs with B cell lymphoma. (DOCX)

S6 Table miRNAs with a significantly different expression in the B cell lymphoma non-remission group (non-responders and dogs that relapsed during CHOP) compared to dogs that completed CHOP in complete remission. (DOCX)

S7 Table miRNAs with a significantly different expression in dogs with B cell lymphoma that died prior to one year compared to dogs that were alive at one year. (DOCX)

S8 Table miRNAs with high versus low miRNA expression significantly correlated with progression-free survival (days). (DOCX)

S9 Table miRNAs with high versus low miRNA expression significantly correlated with overall survival (days). (DOCX)

S1 Data Raw Ct values for 38 canine target miRNAs and controls for all plasma samples. (XLS)

S2 Data Raw Ct values for 38 canine target miRNAs and controls for all lymph node samples. (XLS)
