# Supplementary figures and images for: MicroRNA profiling in canine multicentric lymphoma
Source: PLoS One. 2019 Dec 11;14(12):e0226357. doi: 10.1371/journal.pone.0226357 (PMC6905567; doi:10.1371/journal.pone.0226357)

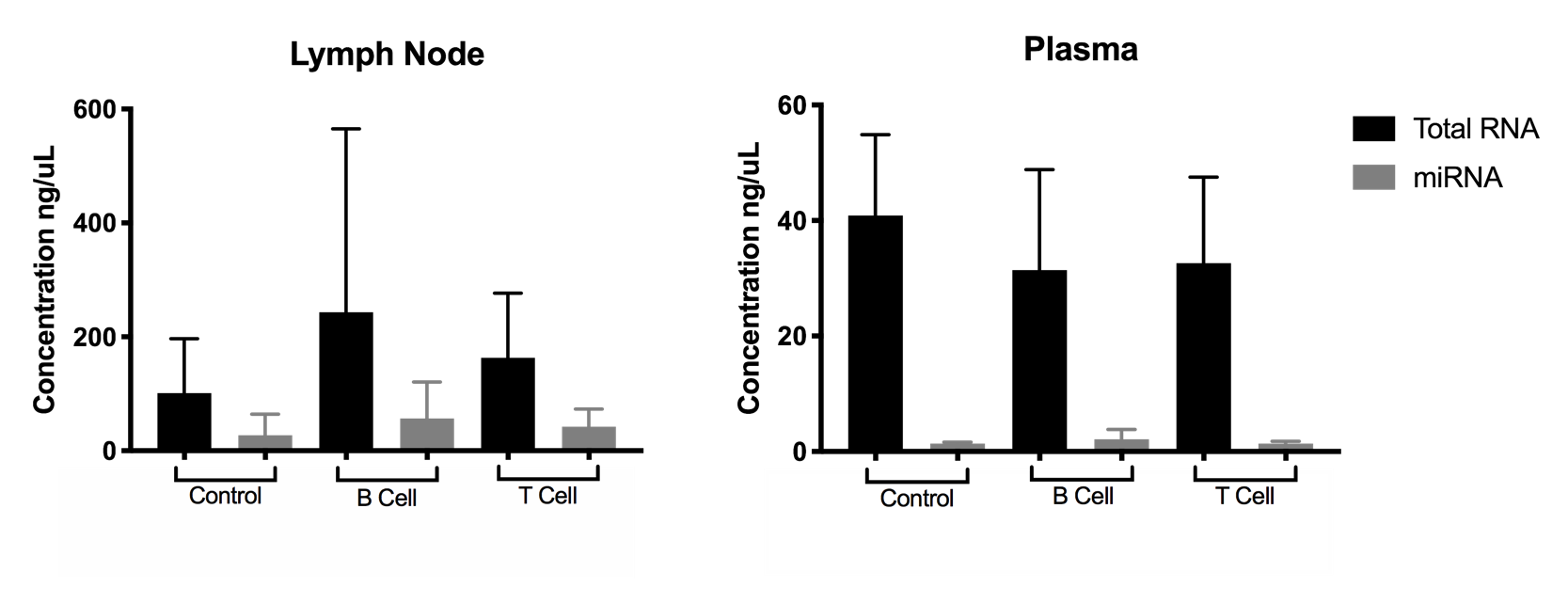

Supplement: S1 Fig — Error bars represent mean +/- standard deviation. (TIF) [file pone.0226357.s001.tif]

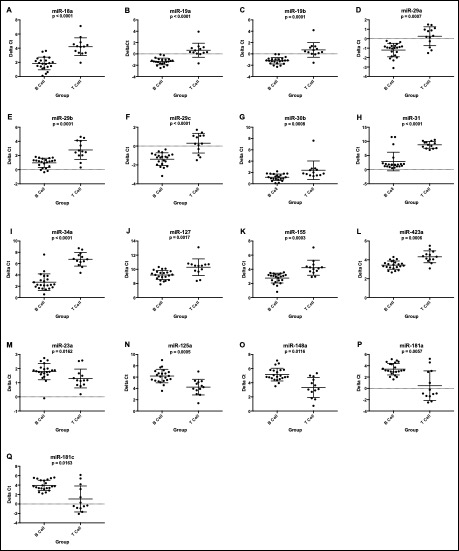

Supplement: S2 Fig — These miRNAs had significantly higher expression in B cell (A-L) or T cell (M-Q) lymphoma compared to the other immunophenotype. (Kruskal-Wallis one-way ANOVA with Dunn’s multiple comparisons test, p-value <0.05; additional group comparisons are shown in Figs 1 and 3). Error bars represent mean +/- standard deviation. (TIF) [file pone.0226357.s002.tif]

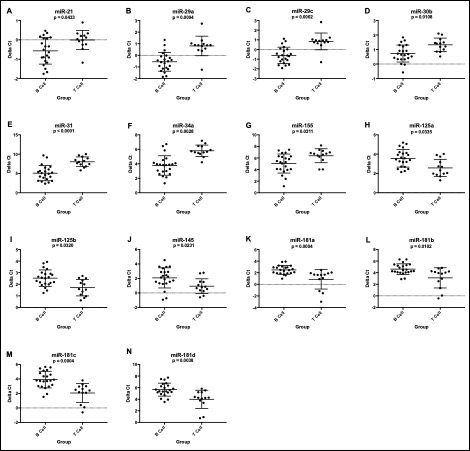

Supplement: S3 Fig — These miRNAs had significantly higher expression in B cell (A-G) or T cell (H-N) lymphoma compared to the other immunophenotype. (Kruskal-Wallis one-way ANOVA with Dunn’s multiple comparisons test, p-value <0.05; additional group comparisons are shown in Figs 3 and 5). Error bars represent mean +/- standard deviation. (TIF) [file pone.0226357.s003.tif]
