# Supplementary material for: MicroRNA profiling in canine multicentric lymphoma
Source: PLoS One. 2019 Dec 11;14(12):e0226357. doi: 10.1371/journal.pone.0226357 (PMC6905567; doi:10.1371/journal.pone.0226357)
Supplement: S1 Table — (DOCX) [file pone.0226357.s004.docx]

S1 Table.

| **Target miR** | **Average delta Ct (Control)** | **Average delta Ct (Lymphoma)** | **Fold change** | **P-value** |
| --- | --- | --- | --- | --- |
| **B cell lymphoma** |  |  |  |  |
| cfa-miR-34a | 6.08 | 2.72 | 10.2519 | 0.0011 |
| cfa-miR-18a | 4.33 | 1.84 | 5.6249 | <0.0001 |
| cfa-miR-130b | 7.75 | 5.67 | 4.2369 | 0.0004 |
| cfa-miR-19a | 0.75 | -1.33 | 4.2219 | <0.0001 |
| cfa-miR-19b | 0.82 | -1.21 | 4.0584 | <0.0001 |
| cfa-miR-423a | 4.48 | 3.44 | 2.0553 | <0.0001 |
| cfa-miR-30b | 1.93 | 1.16 | 1.7060 | 0.0024 |
| cfa-miR-23a | 0.99 | 1.79 | -1.7399 | 0.0006 |
| cfa-miR-26b | 0.26 | 1.08 | -1.7730 | 0.0035 |
| cfa-miR-146a | 1.63 | 2.89 | -2.3972 | 0.0391 |
| cfa-miR-99a | 3.34 | 4.88 | -2.9166 | 0.0019 |
| cfa-miR-145 | 3.09 | 4.70 | -3.0593 | 0.0041 |
| cfa-miR-125b | 2.62 | 4.51 | -3.7188 | 0.0007 |
| cfa-miR-125a | 4.22 | 6.19 | -3.9239 | 0.0008 |
| cfa-miR-150 | -1.86 | 0.84 | -6.5083 | 0.0001 |
| cfa-miR-148a | 1.45 | 5.15 | -13.0206 | <0.0001 |
| **T cell lymphoma** |  |  |  |  |
| cfa-miR-181c | 3.88 | 1.06 | 7.0537 | 0.0452 |
| cfa-miR-182 | 8.37 | 6.04 | 5.0485 | 0.0333 |
| cfa-miR-130b | 7.75 | 5.96 | 3.4589 | 0.0052 |
| cfa-miR-26b | 0.26 | 1.17 | -1.8880 | 0.0005 |
| cfa-miR-21 | -1.83 | -0.80 | -2.0285 | 0.0154 |
| cfa-miR-155 | 3.06 | 4.22 | -2.2425 | 0.0039 |
| cfa-miR-125b | 2.62 | 4.44 | -3.5363 | 0.0041 |
| cfa-miR-150 | -1.86 | 0.03 | -3.6965 | 0.0433 |
| cfa-miR-99a | 3.34 | 5.21 | -3.6487 | 0.0038 |
