# Supplementary material for: MicroRNA profiling in canine multicentric lymphoma
Source: PLoS One. 2019 Dec 11;14(12):e0226357. doi: 10.1371/journal.pone.0226357 (PMC6905567; doi:10.1371/journal.pone.0226357)
Supplement: S2 Table — (DOCX) [file pone.0226357.s005.docx]

S2 Table.

| **Target miR** | **Average delta Ct (B cell)** | **Average delta Ct (T cell)** | **Fold change** | **P-value** |
| --- | --- | --- | --- | --- |
| **B cell lymphoma** |  |  |  |  |
| cfa-miR-31 | 2.87 | 8.77 | 60.0377 | <0.0001 |
| cfa-miR-34a | 2.72 | 6.77 | 16.5695 | <0.0001 |
| cfa-miR-18a | 1.84 | 4.21 | 5.1862 | <0.0001 |
| cfa-miR-19a | -1.33 | 0.65 | 3.9447 | <0.0001 |
| cfa-miR-19b | -1.21 | 0.70 | 3.7569 | <0.0001 |
| cfa-miR-29b | 0.97 | 2.78 | 3.5224 | <0.0001 |
| cfa-miR-29c | -1.39 | 0.30 | 3.2379 | <0.0001 |
| cfa-miR-29a | -1.20 | 0.26 | 2.7639 | 0.0003 |
| cfa-miR-155 | 2.76 | 4.22 | 2.7621 | 0.0022 |
| cfa-miR-30b | 1.16 | 2.39 | 2.3638 | 0.0010 |
| cfa-miR-127 | 9.19 | 10.29 | 2.1474 | 0.0025 |
| cfa-miR-423a | 3.44 | 4.32 | 1.8423 | 0.0033 |
| **T cell lymphoma** |  |  |  |  |
| cfa-miR-181a | 3.40 | 0.48 | 7.5714 | 0.0027 |
| cfa-miR-181c | 3.95 | 1.06 | 7.3753 | 0.0081 |
| cfa-miR-125a | 6.19 | 4.23 | 3.8714 | 0.0002 |
| cfa-miR-148a | 5.15 | 3.33 | 3.5361 | 0.0043 |
| cfa-miR-23a | 1.79 | 1.30 | 1.4039 | 0.0071 |
