# Supplementary material for: MicroRNA profiling in canine multicentric lymphoma
Source: PLoS One. 2019 Dec 11;14(12):e0226357. doi: 10.1371/journal.pone.0226357 (PMC6905567; doi:10.1371/journal.pone.0226357)
Supplement: S3 Table — (DOCX) [file pone.0226357.s006.docx]

S3 Table.

| **Target miR** | **Average delta Ct (Control)** | **Average delta Ct (Lymphoma)** | **Fold change** | **P-value** |
| --- | --- | --- | --- | --- |
| **B cell lymphoma** |  |  |  |  |
| cfa-miR-34a | 6.82 | 3.76 | 8.2939 | <0.0001 |
| cfa-miR-31 | 7.00 | 5.07 | 3.8114 | 0.0240 |
| cfa-miR-182 | 8.29 | 6.71 | 2.9958 | 0.0074 |
| cfa-miR-29b | 4.03 | 2.79 | 2.3528 | 0.0003 |
| cfa-miR-29c | 0.61 | -0.60 | 2.3167 | 0.0107 |
| cfa-miR-29a | 0.58 | -0.55 | 2.1892 | 0.0118 |
| cfa-miR-23a | -2.27 | -1.36 | -1.8686 | 0.0010 |
| cfa-miR-423a | -1.28 | -0.30 | -1.9733 | 0.0188 |
| cfa-miR-181d | 4.44 | 5.65 | -2.3225 | 0.0157 |
| cfa-miR-99a | 1.94 | 3.20 | -2.3935 | 0.0004 |
| cfa-miR-26b | -0.71 | 0.60 | -2.4736 | <0.0001 |
| cfa-miR-143 | 3.85 | 5.17 | -2.4815 | 0.0249 |
| cfa-miR-145 | 0.53 | 2.08 | -2.9150 | 0.0133 |
| cfa-miR-125b | 0.67 | 2.53 | -3.6103 | <0.0001 |
| cfa-miR-125a | 1.10 | 3.56 | -5.4850 | <0.0001 |
| **T cell lymphoma** |  |  |  |  |
| cfa-miR-23a | -2.27 | -1.57 | -1.6212 | 0.0311 |
| cfa-miR-26b | -0.71 | 0.15 | -1.8039 | 0.0365 |
| cfa-miR-423a | -1.28 | -0.19 | -2.1324 | 0.0173 |
