# Supplementary material for: MicroRNA profiling in canine multicentric lymphoma
Source: PLoS One. 2019 Dec 11;14(12):e0226357. doi: 10.1371/journal.pone.0226357 (PMC6905567; doi:10.1371/journal.pone.0226357)
Supplement: S4 Table — (DOCX) [file pone.0226357.s007.docx]

S4 Table.

| **Target miR** | **Average delta Ct (B cell)** | **Average delta Ct (T cell)** | **Fold change** | **P-value** |
| --- | --- | --- | --- | --- |
| **B cell lymphoma** |  |  |  |  |
| cfa-miR-31 | 5.07 | 8.02 | 7.7238 | <0.0001 |
| cfa-miR-34a | 3.76 | 5.57 | 4.0866 | 0.0028 |
| cfa-miR-29c | -0.60 | 0.84 | 2.7127 | 0.0002 |
| cfa-miR-29a | -0.55 | 0.81 | 2.5702 | 0.0004 |
| cfa-miR-155 | 5.04 | 6.39 | 2.5450 | 0.0311 |
| cfa-miR-30b | 0.72 | 1.33 | 1.5205 | 0.0108 |
| cfa-miR-21 | -0.57 | -0.01 | 1.4769 | 0.0433 |
| **T cell lymphoma** |  |  |  |  |
| cfa-miR-181c | 3.91 | 2.06 | 3.5874 | 0.0004 |
| cfa-miR-181d | 5.65 | 3.99 | 3.1718 | 0.0038 |
| cfa-miR-181a | 2.48 | 0.86 | 3.0873 | 0.0004 |
| cfa-miR-181b | 4.61 | 3.10 | 2.8521 | 0.0102 |
| cfa-miR-145 | 2.08 | 0.93 | 2.2094 | 0.0231 |
| cfa-miR-125a | 3.56 | 2.57 | 1.9827 | 0.0335 |
| cfa-miR-125b | 2.53 | 1.71 | 1.7607 | 0.0320 |
