# Supplementary material for: MicroRNA profiling in canine multicentric lymphoma
Source: PLoS One. 2019 Dec 11;14(12):e0226357. doi: 10.1371/journal.pone.0226357 (PMC6905567; doi:10.1371/journal.pone.0226357)
Supplement: S5 Table — (DOCX) [file pone.0226357.s008.docx]

S5 Table.

| **Target miR** | **Average delta Ct (@ Diagnosis)** | **Average delta Ct (@ Relapse)** | **Fold change** | **P-value** |
| --- | --- | --- | --- | --- |
| **Lymph node** |  |  |  |  |
| cfa-miR-127 | 9.15 | 8.20 | 1.9393 | 0.0039 |
| cfa-miR-181a | 3.72 | 3.38 | 1.2648 | 0.0273 |
| cfa-miR-181b | 4.81 | 4.49 | 1.2464 | 0.0391 |
| cfa-miR-15a | 2.42 | 2.16 | 1.1956 | 0.0195 |
| **Plasma** |  |  |  |  |
| cfa-miR-125b | 2.66 | 1.56 | 2.1518 | 0.0195 |
| cfa-miR-30b | 0.78 | 1.15 | -1.2963 | 0.0039 |
| cfa-miR-182 | 7.26 | 8.28 | -2.0342 | 0.0391 |
| cfa-miR-34a | 3.76 | 4.82 | -2.0801 | 0.0039 |
