# Supplementary material for: MicroRNA profiling in canine multicentric lymphoma
Source: PLoS One. 2019 Dec 11;14(12):e0226357. doi: 10.1371/journal.pone.0226357 (PMC6905567; doi:10.1371/journal.pone.0226357)
Supplement: S6 Table — (DOCX) [file pone.0226357.s009.docx]

S6 Table.

| **Target miR** | **Average delta Ct (Remission)** | **Average delta Ct (Non-remission)** | **Fold change** | **P-value** |
| --- | --- | --- | --- | --- |
| **Lymph node** |  |  |  |  |
| cfa-miR-181d | 6.04 | 4.64 | 2.6375 | 0.0055 |
| cfa-miR-181c | 4.34 | 3.01 | 2.5169 | 0.0011 |
| cfa-mir-181a | 3.70 | 2.59 | 2.1572 | 0.0073 |
| cfa-miR-181b | 4.76 | 3.72 | 2.0539 | 0.0304 |
| cfa-miR-29b | 0.58 | 1.52 | -1.9224 | 0.0003 |
| cfa-miR-101 | 1.87 | 2.89 | -2.0238 | 0.0131 |
| cfa-miR-150 | 0.23 | 1.71 | -2.7830 | 0.0028 |
| **Plasma** |  |  |  |  |
| cfa-miR-450b | 9.23 | 7.92 | 2.4730 | 0.0177 |
| cfa-miR-181a | 2.66 | 2.00 | 1.5883 | 0.0387 |
