# Supplementary material for: MicroRNA profiling in canine multicentric lymphoma
Source: PLoS One. 2019 Dec 11;14(12):e0226357. doi: 10.1371/journal.pone.0226357 (PMC6905567; doi:10.1371/journal.pone.0226357)
Supplement: S7 Table — (DOCX) [file pone.0226357.s010.docx]

S7 Table.

| **Target miR** | **Average delta Ct**  **(Alive @1yr)** | **Average delta Ct**  **(Died <1yr)** | **Fold change** | **P-value** |
| --- | --- | --- | --- | --- |
| **Lymph node** |  |  |  |  |
| cfa-miR-181d | 5.84 | 4.65 | 2.2850 | 0.0326 |
| cfa-miR-181c | 4.13 | 3.05 | 2.1116 | 0.0256 |
| cfa-miR-29b | 0.74 | 1.48 | -1.6713 | 0.0159 |
| cfa-miR-150 | 0.39 | 1.83 | -2.7011 | 0.0082 |
