# Supplementary material for: MicroRNA profiling in canine multicentric lymphoma
Source: PLoS One. 2019 Dec 11;14(12):e0226357. doi: 10.1371/journal.pone.0226357 (PMC6905567; doi:10.1371/journal.pone.0226357)
Supplement: S8 Table — (DOCX) [file pone.0226357.s011.docx]

S8 Table.

|  | **Target miR** | **Median survival (Days)** |  | **P-value** |
| --- | --- | --- | --- | --- |
|  |  | High | Low |  |
| **B cell lymphoma** | **Lymph node** |  |  |  |
|  | cfa-miR-150 | 407.5 | 148 | 0.0125 |
|  | cfa-miR-181b | 230.5 | 459 | 0.0240 |
|  | cfa-miR-181d | 177.5 | 459 | 0.0267 |
|  | **Plasma** |  |  |  |
|  | cfa-miR-21 | 265 | 446 | 0.0390 |
|  | cfa-miR-31 | 230.5 | 459 | 0.0074 |
|  | cfa-miR-155 | 230.5 | 407.5 | 0.0322 |
|  | cfa-miR-222 | 279.5 | 459 | 0.0378 |
| **T cell lymphoma** | **Lymph node** |  |  |  |
|  | cfa-miR-101 | 43 | 114 | 0.0177 |
|  | cfa-miR-143 | 113 | 36 | 0.0253 |
|  | cfa-miR-145 | 113 | 36 | 0.0253 |
|  | **Plasma** |  |  |  |
|  | cfa-miR-31 | 50 | 115 | 0.0246 |
|  | cfa-miR-145 | 43 | 114 | 0.0177 |
|  | cfa-miR-150 | 43 | 114 | 0.0177 |
|  | cfa-miR-155 | 43 | 114 | 0.0177 |
|  | cfa-miR-222 | 50 | 115 | 0.0246 |
