# Supplementary material for: MicroRNA profiling in canine multicentric lymphoma
Source: PLoS One. 2019 Dec 11;14(12):e0226357. doi: 10.1371/journal.pone.0226357 (PMC6905567; doi:10.1371/journal.pone.0226357)
Supplement: S9 Table — (DOCX) [file pone.0226357.s012.docx]

S9 Table.

|  | **Target miR** | **Median survival**  **(Days)** |  | **P-value** |
| --- | --- | --- | --- | --- |
|  |  | High | Low |  |
| **B cell lymphoma** | **Lymph node** |  |  |  |
|  | cfa-miR-150 | 600.5 | 246.5 | 0.0386 |
|  | **Plasma** |  |  |  |
|  | cfa-miR-181c | 439.5 | *Undefined | 0.0457 |
|  | cfa-miR-222 | 439.5 | 1052 | 0.0349 |
| **T cell lymphoma** | **Lymph node** |  |  |  |
|  | cfa-miR-222 | 188 | 919 | 0.0216 |

*Survival exceeded 50% at the longest time point, therefore median survival could not be computed.
